# Construction and validation of the prognostic model for patients with neuroendocrine cervical carcinoma: a competing risk nomogram analysis

**DOI:** 10.1186/s12885-021-09104-9

**Published:** 2022-01-03

**Authors:** Ai-Guo Jiang, Xu Cai

**Affiliations:** Department of Obstetrics and Gynecology, Wenling Maternal and Child Health Care Hospital, Wenling, Taizhou, 317500 Zhejiang province China

**Keywords:** Neuroendocrine cervical carcinoma, Competing risk analysis, Nomogram, Prognosis

## Abstract

**Purpose:**

Neuroendocrine cervical carcinoma (NECC) is an uncommon malignancy of the female reproductive system. This study aimed to evaluate cancer-specific mortality and to construct prognostic nomograms for predicting the survival of patients with NECC.

**Methods:**

we assembled the patients with NECC diagnosed between 2004 to 2015 from the Surveillance, Epidemiology, and End Results (SEER) database. Meanwhile, we identified other patients with NECC from the Wenling Maternal and Child Health Care Hospital between 2002 to 2017. Fine and Gray’s test and Kaplan–Meier methods were used to evaluate cancer-specific mortality and overall survival (OS) rates, respectively. Nomograms were constructed for predicting cancer-specific survival (CSS) and OS for patients with NECC. The developed nomograms were validated both internally and externally.

**Results:**

a total of 894 patients with NECC were extracted from the SEER database, then classified into the training cohort (*n* = 628) and the internal validation cohort (*n* = 266). Besides, 106 patients from the Wenling Maternal and Child Health Care Hospital served as an external validation cohort. Nomograms for predicting CSS and OS were constructed on clinical predictors. The validation of nomograms was calculated by calibration curves and concordance indexes (C-indexes). Furthermore, the developed nomograms presented higher areas under the receiver operating characteristic (ROC) curves when compared to the FIGO staging system.

**Conclusions:**

we established the first competing risk nomograms to predict the survival of patients with NECC. Such a model with high predictive accuracy could be a practical tool for clinicians.

**Supplementary Information:**

The online version contains supplementary material available at 10.1186/s12885-021-09104-9.

## Introduction

Neuroendocrine cervical carcinoma (NECC) is a rare neoplasm, making up only 1–5% of all cervical cancers and < 1% of all neuroendocrine tumors [1–3]. In the USA, there are approximately 100–250 cases diagnosed with NECC each year [4]. A large portion of diseases is due to carcinogenic HPV, primarily 18 and 16 subtypes [5]. The histological classification of NECC consists of 4 categories: typical and atypical carcinoid (low-grade with well differentiated), small cell and large cell neuroendocrine carcinoma (high-grade with poorly differentiated or undifferentiated). To date, high-grade NECC was more common and is due to a higher incidence of metastasis than low-grade [3, 6]. Resulting from the rarity of this disease, the optimal treatment of NECC is still uncertain. Current clinical experience is mostly based on multimodal management extrapolated from small cell lung carcinoma. The prognosis of most patients remains dismal even in an early stage of the disease, with a mean OS of 22 to 40 months and 5-year CSS rates less than 30% to 45% [1, 6–9].

At present, the International Federation of Gynecology and Obstetrics (FIGO) staging system is the most common model for predicting survival in patients with NECC [10]. However, the FIGO system only considers the anatomic characteristic of the tumor while ignoring other factors with prognostic values regarding age, race, histological grade, and treatment patterns [1, 11, 12]. Moreover, the prognosis is influenced by many variables with reciprocal influences, while few studies had incorporated all prognostic factors into a predictive system. Therefore, it is necessary to develop an accurate prediction model to evaluate the prognoses of NECC patients. The nomogram is a convenient prognostic tool to calculate the survival outcomes, which can help clinicians making personalized options for patients through an intuitive graphic model [13]. It has been widely used in various neuroendocrine tumors and proven to be effective [14, 15].

As cancer progression, several comorbidities have occurred with increasing age. For example, older patients are more likely to face cardiovascular disease, liver disease, and metabolic diseases than the younger ones. Deaths caused by these non-cancer comorbidities have become the competing events of NECC [16]. Failure to evaluate such risks would make incorrect conclusions [17]. Besides, consideration of competing risks can correspond to the informative character of censoring and independently calculate the incidence of each factor [18]. Most previous studies were based on the Kaplan–Meier method and Cox regression hazard model, which may overestimate the portion of cancer-specific death and prolong the follow-up time [19]. Therefore, traditional methods are inappropriate and should be replaced by the competing risk model. However, for all we know, a competing risk nomogram for NECC is yet to be reported. Based on the Surveillance, Epidemiology, and End Results (SEER) database, the objective of this study was to construct competing nomograms for predicting CSS and OS in patients with NECC.

## Materials and methods

### Study cohorts

We identified patients diagnosed with NECC between 2004 and 2015 from the SEER-18 database, using SEER*Stat software (version 8.3.6; National Cancer Institute, USA). The SEER database collects cancer information of 18 registries in the US and accounts for approximately 28% of the US population, which offers considerable data for detail analysis [20, 21]. Depending on the International Classification of Diseases for Oncology, Third Edition (ICD-O-3), Cases were selected based on the primary site code (C53.0-C53.1, C53.8-C53.9) and associated histology codes (8010–8053). All eligible patients were those who had only one primary malignancy, complete clinicopathological information, and full follow-up results. The exclusion criteria included (1) multiple tumors, (2) diagnosed only with clinical manifestation or radiography, lack of important clinical pathology result (3) clinical information missed or unknown, (4) survival data were unavailable or incomplete. Besides, we retrieved information of patients with NECC who met the same criteria at the department of gynecology and obstetrics of Wenling Maternal and Child Health Care Hospital from 2002 to 2017 using medical management systems, and all participated patients are informed consent. Patients from the SEER database were randomly assigned to the training set and the internal validation set in a 7:3 split ratio. Patients from our database served as an independent external validation set. Our study had received the approval of the Institutional Review Board of Wenling Maternal and Child Health Care Hospital, and we had access to the SEER program data after obtaining permission from the US National Cancer Institute (username number: 17620-Nov2018).

### Data collection

Demographic and clinical variables, including age, year at diagnosis, marital status, race, histology grade, FIGO stage, tumor size, distant metastasis, treatment strategy, vital status, cause of death, and survival time were extracted and analyzed. We utilized the X-tile program (Yale University, New Haven, USA) to obtain optimal cut-off points then divided patients age into three groups: < 44 years, 44—67 years, > 67 years (Figure S1). Subsequently, we also classified patients with tumor size: < 2 cm, 2—4 cm, and > 4 cm. In our study, “Married” was defined as married or unmarried but having domestic partner, while widowed, divorced, and separated were recorded as “others”. Metastasis defined as lymphatic or distant organ metastasis. Surgery and radiotherapy referred to local treatment for the primary tumor. OS and CSS were calculated as the period from diagnosis to death owing to any causes or NECC, respectively. Cases were censored if alive at last follow-up.

**Fig. 1:**
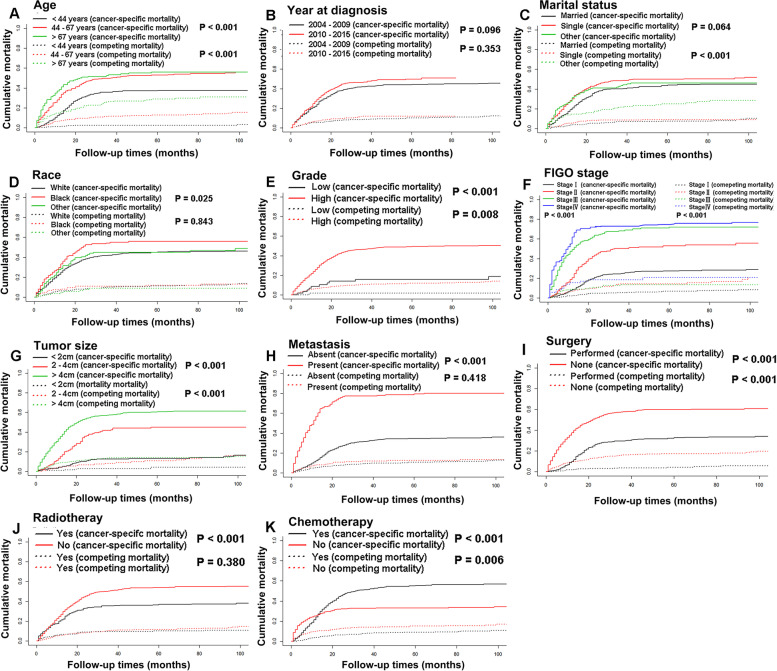
The cumulative incidence function curves for cancer-specific mortality and competing mortality based on patient characteristics: age (**A**); year at diagnosis (**B**); marital status (**C**); race (**D**); grade (**E**); FIGO stage (**F**); tumor size (**G**); metastasis (**H**); surgery (**I**); radiotherapy (**J**); chemotherapy (**K**)

### Statistical analysis

We treated death from cancer and death from non-cancer as two competing events. The cumulative incidence function (CIF) was estimated using the Fine and Gray’s test to compare the single-factor incidence of each competing event at different time points (1-, 3-, and 5-year). The proportional sub-distribution hazard model was used to identify the significant variables of CSS, and competing risk nomogram was developed based on these predictors. OS rates were evaluated using the Kaplan–Meier method and compared using the Log-rank test. Prognostic variables identified from the multivariate Cox analysis were combined to construct the nomogram for OS.

The performance of nomograms was measured by concordance indexes (C-indexes) and calibration curves. A C-index greater than 0.7 indicates a relatively strong discrimination ability. The calibration plots were applied using a bootstrap approach with 1000 resamples to show the consistency between observation and prediction. Furthermore, prognostic precision comparisons between the developed nomograms and the FIGO staging system were visualized by the receiver operating characteristic (ROC) curves and quantified by the area under the ROC curve (AUC) values. All the statistical analysis was conducted using R software version 3.6.1. The P-value of two-sided < 0.05 was deemed statistically significant.

## Results

### Patients baseline characteristics

A total of 894 eligible patients with NECC were assembled from the SEER database and assigned to the training cohort (*n* = 628) and the internal validation cohort (*n* = 266) (Table 1). Based on the same inclusion criteria, another 106 patients with NECC from our dataset were treated as the external validation cohort (Table S1). Of the total SEER group, there was no statistical distinction in the demographic and clinical characteristics between the two subgroups. A substantial portion of patients (*n* = 749, 83.8%) were under 67 years old, and most patients were married status (*n* = 401, 44.9%). Regarding to the disease features, the majority of patients were high grade (*n* = 836, 93.5%), stage I (*n* = 411, 46.0%), greater than 4 cm in tumor size (*n* = 526, 58.8%), and absent of metastasis (*n* = 643, 71.9%). Concerning the treatment strategy, more than half patient received chemotherapy (*n* = 577, 64.5%), and did not have surgery (*n* = 481, 53.8%) and radiotherapy (*n* = 547, 61.2%).

**Table 1:** Demographics and Clinicopathologic Characterisitcs of SEER Patients With cNET

**Cetegory**	**Trainning cohort (*n* = 628)**	**Internal validation cohort (*n* = 266)**	**Total cohort (*n* = 894)**	***P***
	No. of patients (%)	No. of patients (%)	No. of patients (%)	
**Age(year)**				0.498
< 44	(42.7%) 104	104 (39.1%)	372 (41.6%)	
44–67	263 (41.9%)	114 (42.9%)	377 (42.2%)	
> 67	97 (15.4%)	48 (18.0%)	145 (16,2%)	
**Year at diagnosis**				0.188
2004–2009	294 (46.8%)	138 (51.9%)	432 (48.3%)	
2010–2015	334 (53.2%)	128 (48.1%)	462 (51.7%)	
**Marital status**				0.901
Married	281 (44.7%)	120 (45.1%)	401 (44.9%)	
Single	247 (39.4%)	101 (38.0%)	348 (38.9%)	
Other	100 (15.9%)	45 (16.9%)	145 (16.2%)	
**Race**				0.275
White	456 (72.6%)	180 (67.7%)	636 (71.1%)	
Black	101 (16.1%)	47 (17.6%)	148 (16.6%)	
Other^a^	71 (11.3%)	39 (14.7%)	110 (12.3%)	
**Grade**^**b**^				0.208
Low	44 (7.0%)	14 (5.3%)	58 (6.5%)	
High	584 (93.0%)	252 (94.7%)	836 (93.5%)	
**Stage**				0.501
I	298 (47.5%)	113 (42.5%)	411 (46.0%)	
II	143 (22.8%)	69 (25.9%)	212 (23.7%)	
III	136 (21.6%)	64 (24.1%)	200 (22.4%)	
IV	51 (8.1%)	20 (7.5%)	71 (7.9%)	
**Tumor size**				0.726
< 2 cm	142 (22.6%)	54 (20.3%)	196 (21.9%)	
2–4 cm	121 (19.3%)	51 (19.2%)	172 (19.2%)	
> 4 cm	365 (58.1%)	161 (60.5%)	526 (58.8%)	
**Metastasis**				0.625
Absent	455 (72.5%)	188 (70.7%)	643 (71.9%)	
Present	173 (27.5%)	78 (29.3%)	251 (28.1%)	
**Surgery**				0.714
Performed	293 (46.7%)	120 (45.1%)	413 (46.2%)	
None	335 (53.3%)	146 (54.9%)	481 (53.8%)	
**Radiotherapy**				0.653
Yes	247 (39.3%)	100 (37.6%)	347 (38.8%)	
No	381 (60.7%)	166 (62.4%)	547 (61.2%)	
**Chemotherapy**				0.917
Yes	406 (64.6%)	171 (64.3%)	577 (64.5%)	
No	222 (35.4%)	95 (35.7%)	317 (35.5%)	

^a^American Indian/Alaskan Native, Asian/Pacific Islander, ^b^Low: Grade I (well differentiated) and Grade II (moderately differentiated); High: Grade III (poorly differentiated) and Grade IV (undifferentiated anaplastic)

In the SEER group, the median follow-up duration was 20 months (ranges, 1–155 months). A total of 504/894 (56.4%) deaths occurred during follow-up, of which 401 deaths were ascribed to NECC, and 103 died from competing events. The 1, 3, and 5-year cancer-specific mortality were 24.6, 44.3, 46.9%, respectively. As showed in Fig. 1, CIF curves indicated that patients with characteristics of older age, higher grade, more advanced stage, larger tumor size, and no surgical treatment were all likely to die from both NECC and competing events. Patients with characteristics of black people, the absence of radiotherapy, the presence of metastasis, and receiving chemotherapy were observed with increasing cumulative mortality from NECC, while not associated with competing causes. There is no statistical difference in cancer-specific mortality of the characteristics regarding marital status and year at diagnosing (Table 2).

**Table 2:** Overall survival rates and cumulative incidences of mortality among SEER patients with cNET

Characteristic	Patients		Overall survival rate (%)			*P*	Cancer-specific mortality (%)			*P*	Non-cancer-specific mortality (%)			*P*
	**No**	**(%)**	**1 year**	**3 year**	**5 year**		**1 year**	**2 year**	**3 year**		**1 year**	**2 year**	**3 year**	
**Total**	894	100	69.2	45.5	41.9		24.6	44.3	46.9		6.1	10.1	11.1	
**Age(years)**														
** < **44	372	41.6	85.0	61.8	60.0	< 0.001	13.6	35.7	37.5	< 0.001	1.3	2.5	2.6	< 0.001
45–67	377	42.2	62.5	38.9	34.4		30.2	49.5	52.9		7.2	11.5	12.7	
> 67	145	16.2	46.5	20.8	15.2		38.2	53.1	55.9		15.2	26.1	28.8	
**Year**														
2004–2009	432	48.3	72.5	49.4	45.5	0.025	22.1	41.9	44.4	0.096	5.3	8.6	10.1	0.353
2010–2015	462	51.7	66.1	41.4	38.4		27.1	46.7	49.7		6.8	11.8	11.9	
**Marital Status**														
Married	348	38.9	77.3	52.7	48.4	< 0.001	19.1	40.2	43.9	0.064	3.4	7.1	7.5	< 0.001
Single	401	44.9	66.4	42.8	41.2		28.3	48.6	49.9		5.3	8.5	8.9	
Other	145	16.2	57.7	35.6	28.9		27.7	42.7	46.1		14.5	21.6	25.1	
**Race**^**a**^														
White	636	71.1	70.8	47.9	43.6	0.002	22.9	41.9	45.1	0.025	6.2	10.2	11.3	0.843
Black	148	16.6	62.8	34.7	32.1		29.8	54.3	56.0		7.4	10.9	11.9	
Other^1^	110	12.3	68.7	46.1	45.4		27.6	45.0	45.2		3.6	8.8	8.9	
**Grade**^**b**^														
Low	58	6.5	89.7	82.4	80.5	< 0.001	8.6	15.8	15.9	< 0.001	1.7	1.8	1.9	0.008
High	836	93.5	67.8	42.8	38.9		25.8	46.4	49.2		6.4	10.7	11.8	
**FIGO Stage**														
I	411	46.0	89.1	70.0	65.7	< 0.001	8.4	24.6	27.3	< 0.001	2.4	5.3	6.9	< 0.001
II	212	23.7	72.2	35.4	32.9		20.6	50.3	52.8		7.1	14.2	14.3	
III	200	22.4	42.0	18.4	14.5		48.9	68.2	72.1		9.1	13.4	13.5	
IV	71	7.9	22.5	4.8	0		61.9	76.7	76.8		15.5	18.4	10.8	
**Tumor Size**														
< 2	196	22.0	94.8	84.9	82.8	< 0.001	4.1	12.4	13.1	< 0.001	1.1	2.6	4.1	0.001
2–4	172	19.2	80.7	50.3	44.5		15.2	41.5	44.8		4.1	8.1	10.6	
> 4	526	58.8	56.0	28.9	25.4		35.3	57.4	60.6		8.6	13.6	13.9	
**Metastasis**														
Absent	643	71.9	82.4	58.8	54.8	< 0.001	12.2	31.6	34.5	< 0.001	5.3	9.5	10.6	0.418
Present	251	28.1	35.3	11.0	8.2		56.6	77.2	79.3		8.1	11.7	12.4	
**Surgery**														
Performed	413	46.2	88.2	67.1	63.1	< 0.001	9.8	29.3	32.5	< 0.001	1.9	3.5	4.2	< 0.001
None	481	53.8	52.9	26.4	23.1		37.4	57.5	59.6		9.6	16.1	17.2	
**Radiotherapy**														
Yes	347	38.8	72.1	55.3	52.9	< 0.001	21.2	35.2	36.7	< 0.001	6.6	9.4	10.2	0.380
No	547	61.2	67.4	38.8	34.3		26.8	50.4	53.9		5.7	10.6	11.7	
**Chemotherapy**														
Yes	577	64.5	71.9	40.6	35.7	0.018	24.2	51.2	55.4	< 0.001	3.8	8.1	8.9	0.006
No	317	35.5	64.4	53.3	51.8		25.4	32.7	33.1		10.2	13.9	15.1	

^a^American Indian/Alaskan Native, Asian/Pacific Islander, ^b^Low: Grade I (well differentiated) and Grade II (moderately differentiated); High: Grade III (poorly differentiated) and Grade IV(undifferentiated anaplastic)

**Fig. 2:**
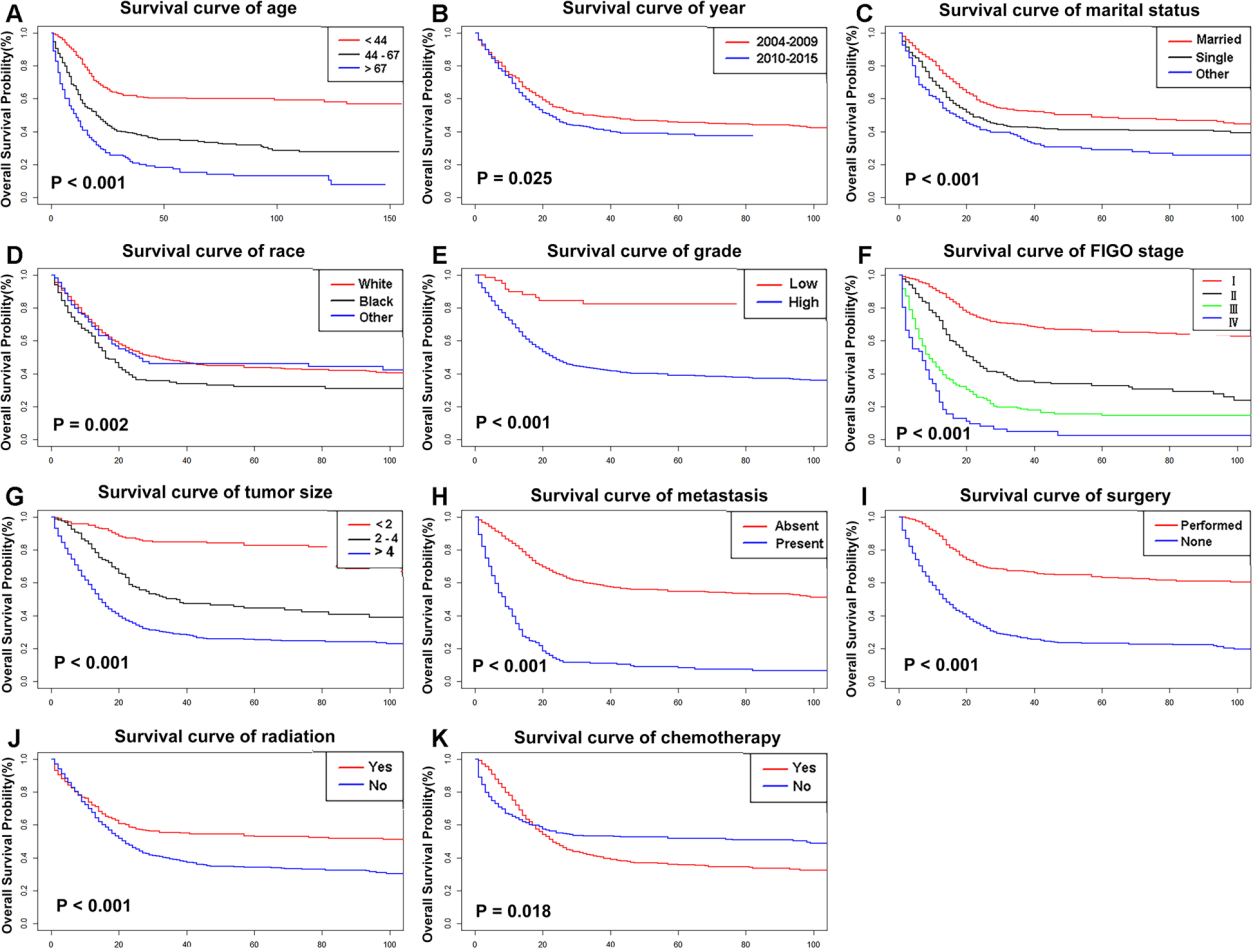
The survival curves for OS rates based on patient characteristics: age (**A**); year at diagnosis (**B**); marital status (**C**); race (**D**); grade (**E**); FIGO stage (**F**); tumor size (**G**); metastasis (**H**); surgery (**I**); radiotherapy (**J**); chemotherapy (**K**)

For OS, the 1-, 3-, and 5-year survival rates were 69.2, 45.5, 41.9%, respectively. The survival curves of OS based on each variable were shown in Fig. 2. Variables regarding with older age, diagnosed after 2010, divorced or widowed status, black race, higher grade, advanced stage, enlarged tumor, the presence of metastasis, and receiving chemotherapy all had an inferior OS, while patients who underwent surgery and radiotherapy had a better OS (Table 2).

### Independent predictors of patients with NECC

Factors including age, marital status, grade, stage, tumor size, metastasis, surgery, radiotherapy, and chemotherapy were significantly related to CSS through univariate competing analysis. While age, year at diagnosis, marital status, histology grade, FIGO stage, tumor size, distant metastasis, surgery, radiotherapy, and chemotherapy were significantly associated with OS through univariate analysis. The race was not a risk factor for both CSS and OS (Table 4). The variables identified from the univariate analysis were further analyzed by multivariate analysis of CSS and OS (Table 3). After adjusting the confounding factors, multivariate analysis revealed that age, FIGO stage, tumor size, metastasis, and chemotherapy were independent predictors for both CSS and OS (Table 4).

**Table 3:** Univariate analysis for OS and CSS in training cohort

**Cetegory**	**Overall survival**	**Cancer-specific survival**
	***P* (Log-rank test)**	***P* (grey's test)**
**Age(year)**		
< 44 vs. 44–67	< 0.001	< 0.001
< 44 vs. > 67	< 0.001	< 0.001
**Year at diagnosis**		
2004–2009 vs. 2010–2015	0.023	0.076
**Marital status**		
Married vs. Single	0.009	0.012
Married vs. Other	< 0.001	0.031
**Race**		
White vs. Black	0.025	0.034
White vs. Other^a^	0.549	0.264
**Grade**^**b**^		
Low vs. High	< 0.001	< 0.001
**Stage**		
I vs II	< 0.001	< 0.001
I vs III	< 0.001	< 0.001
I vs IV	< 0.001	< 0.001
**Tumor size**		
< 2 cm vs. 2–4 cm	< 0.001	< 0.001
< 2 cm vs. > 4 cm	< 0.001	< 0.001
**Metastasis**		
Absent vs. Present	< 0.001	< 0.001
**Surgery**		
Performed vs. None	< 0.001	< 0.001
**Radiotherapy**		
No	< 0.001	< 0.001
**Chemotherapy**		
Yes vs. No	0.034	< 0.001

^a^American Indian/Alaskan Native, Asian/Pacific Islander, ^b^Low: Grade I (well differentiated) and Grade II (moderately differentiated, High: Grade III (poorly differentiated) and Grade IV (undifferentiated anaplastic).

**Table 4:** Multivariate analysis for OS and CSS in training cohort

**Cetegory**	**Overall survival**	***P***	**Cancer-specific survival**	***P***
	**Hazard Ratio (95% CI)**		**Hazard Ratio (95% CI)**	
**Age(year)**				
< 44	Reference		Reference	
44–67	1.372 (1.051–1.790)	0.019	1.285 (0.964–1.713)	0.086
> 67	1.738 (1.209–2.498)	0.002	1.659 (1.091–2.524)	0.018
**Year at diagnosis**				
2004–2009	Reference		NI	
2010–2015	1.081 (0.864–1.353)	0.492	NI	NI
**Marital status**				
Married	Reference		Reference	
Single	1.043 (0.816–1.332)	0.738	1.104 (0.846–1.440)	0.464
Other	1.207 (0.861–1.691)	0.274	0.916 (0.608–1.381)	0.677
**Grade**^**a**^				
Low	Reference		Reference	
High	1.749 (0.912–3.356)	0.092	1.501 (0.753–2.989)	0.248
**Stage**				
I	Reference		Reference	
II	1.630	0.002	1.545 (1.081–2.209)	0.016
III	2.256	< 0.001	2.403 (1.665–3.467)	< 0.001
IV	2.760	< 0.001	2.652 (1.648–4.628)	< 0.00
**Tumor size**				
< 2 cm	Reference		Reference	
2–4 cm	2.487 (1.512–4.092)	< 0.001	2.127 (1.213–3.732)	0.008
> 4 cm	3.104 (1.916–5.028)	< 0.001	2.763 (1.609–4.744)	< 0.001
**Metastasis**				
Absent	Reference		Reference	
Present	2.778 (2.151–3.587)	< 0.001	3.029 (2.293–4.001)	< 0.001
**Surgery**				
Performed	Reference		Reference	
None	1.173 (0.881–1.561)	0.274	0.955 (0.702–1.298)	0.769
**Radiotherapy**				
Yes	Reference		Reference	
No	1.083 (0.831–1.410)	0.556	1.224 (0.910–1.648)	0.180
**Chemotherapy**				
Yes	Reference		Reference	
No	1.979 (1.485–2.637)	< 0.001	1.489 (1.059–2.095)	0.022

*CI* confidence interval, ^a^Low: Grade I (well differentiated) and Grade II (moderately differentiated); High: Grade III (poorly differentiated) and Grade

IV (undifferentiated anaplastic)

### Construction and validation of nomograms

**Fig. 3:**
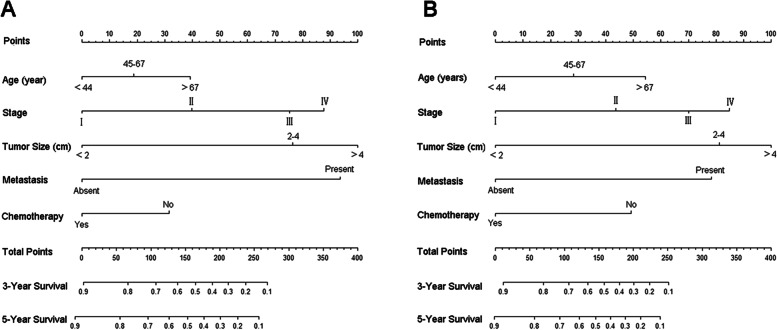
Nomograms for predicting the 3- and 5-year CSS (**A**) and OS (**B**)

**Fig. 4:**
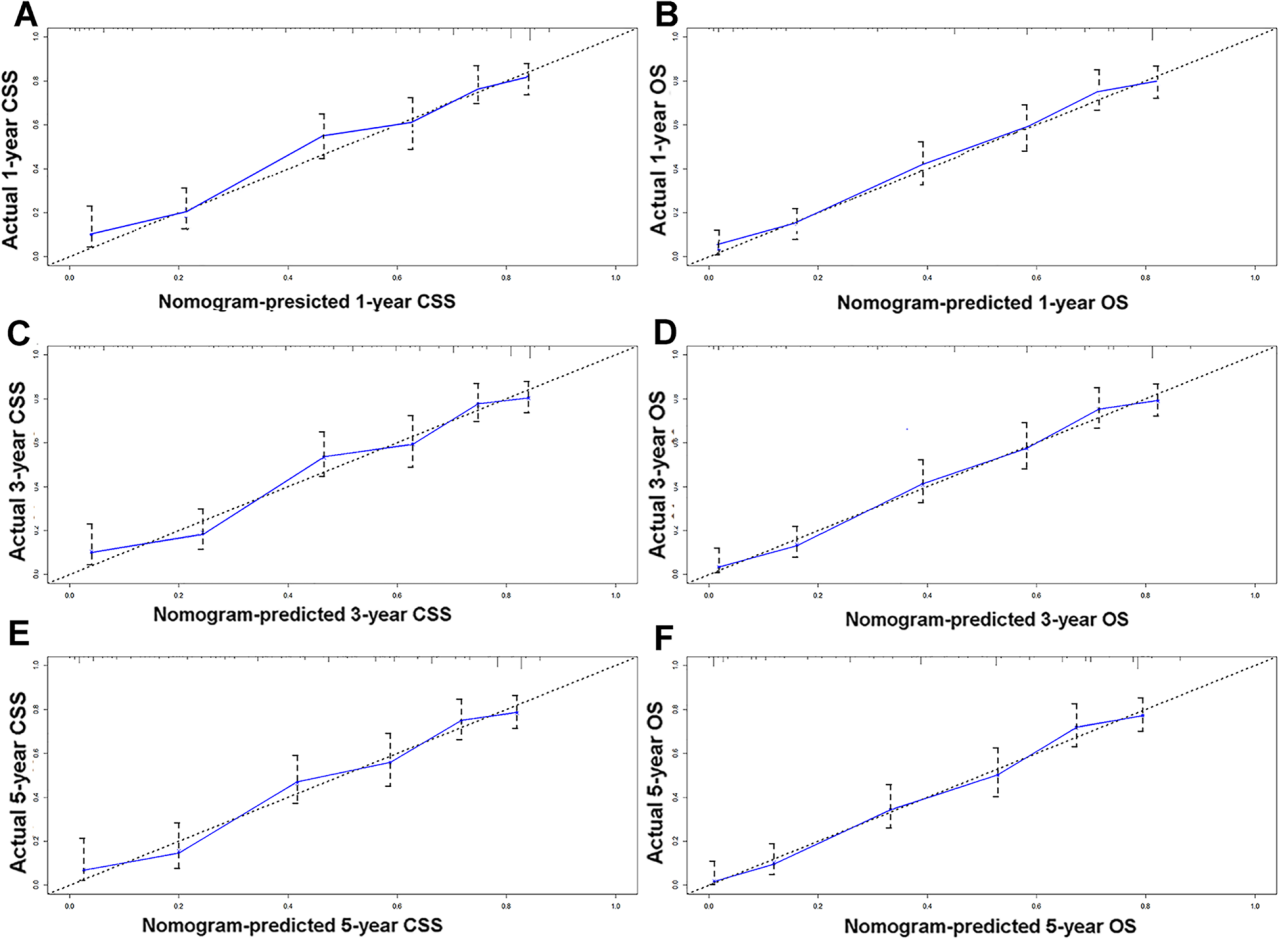
The calibration curves of training cohort show the nomograms-predicted rates (X-axis) are correspondent with actual survival rates (Y-axis), including 1-year CSS (**A**) and OS (**B**), 3-year CSS (**C**) and OS (**D**), 5-year CSS (**E**) and OS (**F**)

**Fig. 5:**
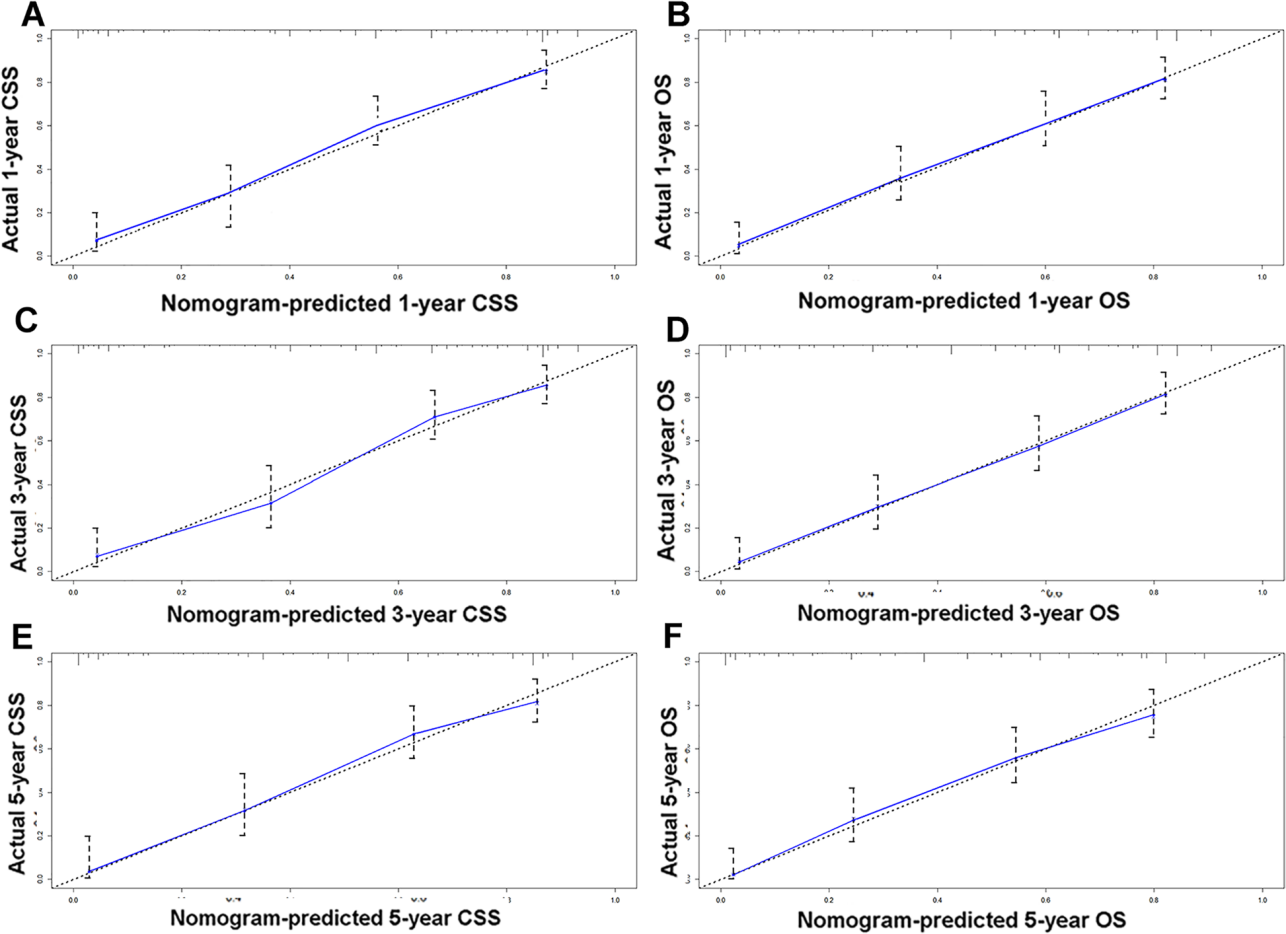
The calibration curves of internal validation cohort show the nomograms-predicted rates (X-axis) are correspondent with actual survival rates (Y-axis), including 1-year CSS (**A**) and OS (**B**), 3-year CSS (**C**) and OS (**D**), the 5-year CSS (**E**) and OS (**F**)

The nomograms for CSS and OS were constructed based on incorporating five prognostic variables from the training cohort. As shown in Fig. 3, tumor size contributed most while chemotherapy accounted for the least contribution to CSS and OS. By summing up the specific point of each predictor then measuring the total points to the CSS and OS, the individual survival probability can be calculated easily. The nomograms were validated internally and externally and indicated an excellent predictive ability. The C-indexes of nomograms in the training cohort for CSS and OS were 0.784 (95% CI: 0.758–0.809), and 0.787 (95% CI, 0.765–0.808), respectively. Additionally, based on the internal and external cohort, the C-indexes of nomograms were also presented more powerful discrimination than those of the FIGO stage (Table 5. The calibration curves of each group revealed a prominent consistency between prediction and observation (Figs. 4 and 5 showed the results of training and internal validation cohort, respectively, and Figure S2 showed the results of external validation cohort).

**Table 5:** C-indexes for the Nomogram and FIGO stage systems in patients with cNET

Survival		Training cohort	*P*	Internal validation cohort	*P*	External validation cohort	*P*
Overall survival	Nomogram	0.787 (95%CI, 0.765–0.808)	< 0.001	0.789 (95%CI, 0.764–0.822)	< 0.005	0.761 (95%CI, 0.702–0.819)	< 0.001
	FIGO stage	0.711 (95%CI, 0.685–0.736		0.724 (95%CI, 0.684–0.763)		0.723 (95%CI, 0.664–0.781)	
Cancer -specific surviva	Nomogram	0.784 (95%CI, 0.758–0.809)	< 0.001	0.798 (95%CI, 0.758–0.837)	< 0.005	0.770 (95%CI, 0.707–0.832)	< 0.001
	FIGO stage	0.709 (95%CI, 0.681–0.736)		0.734 (95%CI, 0.691–0.773)		0.737 (95%CI, 0.674–0.799)	

*CI* confidence interval, *FIGO* the International Federation of Gynecology and Obstetrics

### Comparison with the FIGO staging system

**Fig. 6:**
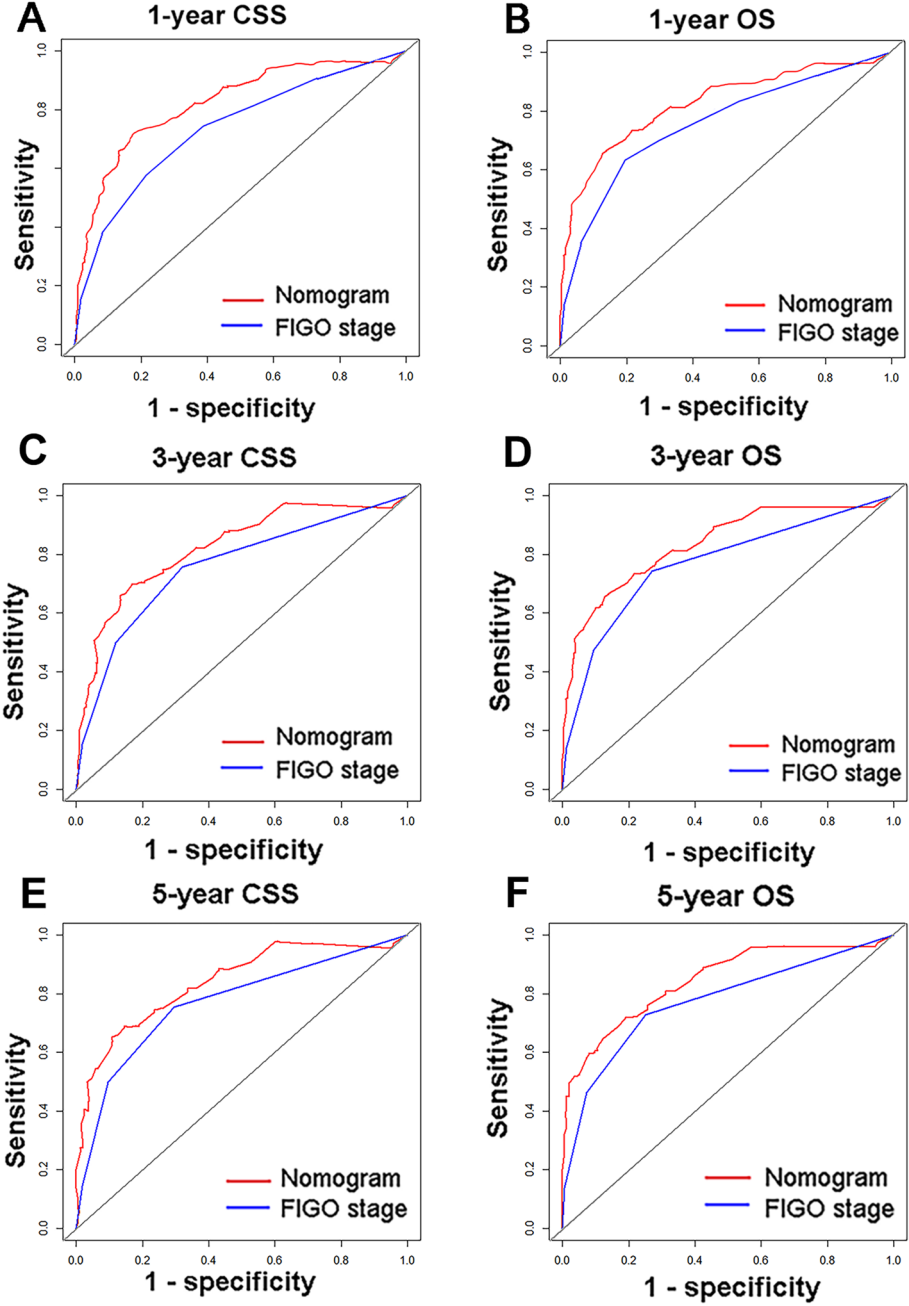
Comparison of the ROC curves for prediction ability between the nomograms and the FIGO staging system, including 1-year CSS (**A**) and OS (**B**), 3-year CSS (**C**) and OS (**D**), and the 5-year CSS (**E**) and OS (**F**)

Compared against the FIGO stage, established nomograms had a relatively higher discrimination ability (Fig. 6). The AUC values of nomograms in the training cohort for 3- and 5-year OS rates were 0.836 and 0.845, respectively, while the AUC values of the FIGO stage for those were 0.769 and 0.711, respectively. Likewise, the AUC values of nomograms for 3- and 5-year CSS rates were higher than those of the FIGO staging system. For the internal and external validation cohort, the similar results were shown in Table 6.

**Table 6:** Comparison of AUC values between Nomogram and FIGO stage

Patients		Overall surviva		Cancer-specific survival	
		**3 year**	**5 year**	**3 year**	**5 year**
**Training cohort**	Nomogram	0.836	0.845	0.822	0.838
	FIGO stage	0.769	0.771	0.758	0.769
**Internal validation cohort**	Nomogram	0.861	0.854	0.875	0.877
	FIGO stage	0.773	0.760	0.793	0.789
**External validation cohort**	Nomogram	0.832	0.833	0.827	0.826
	FIGO stage	0.770	0,774	0.791	0.786

*FIGO* the International Federation of Gynecology and Obstetrics

## Discussion

Several studies have analyzed the prognosis of NECC. However, such data were mainly were primarily small sample sizes [1, 3, 8, 11]. Predictors of NECC are still limited, and efficient treatments to prolong survival for patients are also elusive [20]. In the present study, we first evaluated the cancer-specific mortality and established practical nomograms to predict the survival outcomes of patients with NECC. The developed nomograms were built on selected patients from the SEER database, revealing ideal differentiation and calibration both internally and externally. Based on the hazard ratio in the competing risk model, we determined that factors in terms of stage, tumor size, and metastasis were the most important prognostic factors, which should be the important consideration in making clinical decisions. Moreover, higher AUC values of nomograms demonstrated that nomograms have a superior prediction ability than the FIGO staging system.

In previous studies, age was commonly recognized as an independent predictor of NECC. Based on the National Cancer Database analysis, Benjamin et al. concluded that patients with NECC, compared to patients with squamous cell cervical cancer, were more likely diagnosed before 30 years old and tended to observe adverse survival as increasing age [2]. In another retrospective study, Reem et al. suggested that younger age (< 40 years) was significantly related to improved survival, with a 3-year PFS of 80% versus 40.9% (*p* = 0.044) [22]. In current data, we indicated that 44 and 67 years might be the best cut-off points to make a precise grouping for OS and CSS of NECC patients. Furthermore, the present study also indicated that older patients were at higher risk of competing risk of death. Thus, it is required to take competing event into consideration for predicting cancer-related prognoses.

Our study showed that stage and metastasis are the most prognostic variables for OS and CSS of patients with NECC, which is consistent with prior retrospective reports on a large size cohort [1, 23]. In current data, the most patient diagnosed NECC were early-stage while had poor survival. Similarly, Lee et al. reported that 67.2% of 61 patients were diagnosed at early-stage, with a 5-year survival of only 42.1% [7]. In a recent multi-center study of 193 patients with NECC [24], the risk of progression or death increased with advanced-stage (stage I vs. stage IV, HR = 8.98, 95% CI: 5.25–14.49). NECC tended to widespread hematogenous and lymphatic metastasis despite aggressive therapies [25]. Nagao et al. reported that the most sites of metastasis at primary diagnosis were pelvic lymph, followed by liver, bone, and breast [9]. Tumor size has also been considered as an independent predictor of NECC [7, 26]. We found that most tumors were larger size, and 2 cm was a cut-off for decreased OS and CSS. A previous study, including 115 NECC patients, documented that 80% of patients had tumor ≥ 2 cm while had only 28.6% for 5-year CSS [1]. A meta-analysis containing 1901 patients further confirmed larger tumor size was associated with poor OS (> 4 cm vs. ≤ 4 cm, HR = 1.76; > 2 cm vs. ≤ 2 cm, HR = 1.61) [26].

Currently, there is still no consensus on the first line therapeutic regimen for NECC, and multimodality treatment is advocated [19, 20, 27]. Our data showed that surgery and radiotherapy were associated with improved survival, but they failed to be prognostic predictors in multivariate analysis. Another prior SEER database analysis revealed that radical surgery and primary radiation yielded equally poor survival (5-year OS rates were: 25% vs. 32%, *p* = 0.636) [28]. In China, radical hysterectomy-based surgery followed by adjuvant therapy is the primary treatment for patients with NECC in early FIGO stages [29]. On the contrary, a multi-center study consisted that primary radiotherapy followed by chemotherapy was associated with better survival than primary surgical treatment for early-stage patients (5-year OS rates were 78% vs. 46%, *P* = 0.046) [30]. Whether the use of surgery for locally advanced-stage NECC patients remains unclear [19]. For cervical cancer, it has been widely recognized that post-radiation hysterectomy did not improve survival while risking the injury of the urinary tract. However, Pauline et al. suggested that intensive primary chemoradiotherapy for such patients can enable them to benefit from surgery, with complete surgical margins and improved prognosis [31].

In our study, survival curves of OS demonstrated that performing chemotherapy was associated with poor survival. However, it was found to be independent predictors after adjusting confounding factors in multivariate analysis. This can partly explain more prone to chemotherapy with elderly or metastatic patients, due to low tolerability or unacceptance to surgery. The commonly used chemotherapy regimens were TPB (topotecan, paclitaxel, and bevacizumab) or EP (etoposide and cisplatin) [5]. Other tested regimes include VAC (vincristine, adriamycin, and cyclophosphamide), CPT-P (irinotecan, platinum), and TC (taxane, platinum) [32, 33]. A large cohort study by Cohen et al. [1] reported that the use of platinum-based chemotherapy or chemoradiation was associated with better survival in NECC patients (OR = 0.62, 95%CI:0.41–0.92, *P* = 0.019). For recurrent NECC, targeted therapies and immunologic inhibitors, such as bevacizumab, nivolumab, and trametinib, might be the new options [34–36]. However, patients with NECC have a poor survival irrespective of the aggressive schemes used. The development of novel drugs for NECC is urgently needed.

Based on the SEER database, we conducted the competing risk model and predicted survival outcomes for patients with NECC. These developed nomograms include easily obtained variables from clinical practice, which can help clinicians with access to patients’ counseling and decision-making. The C-index and calibration plots of nomograms at different time points show excellent performance in prediction ability. Furthermore, compared with the traditional FIGO staging system, this current model displays more accuracy in survival outcomes.

There are several limitations to our study. First, selection bias might be unavoidable because of the nature of the retrospective study. Second, some prognostic information, such as invasion depths, lymph-vascular invasion, and biologic markers, could not be included. Besides, more-specific data such as the dose, type, and the course of treatment of adjuvant therapy were unavailable in the SEER database, which could restrict further analysis. Third, although a single-center cohort validation was applied in this study, prospective external validation is still required.

## Conclusion

We constructed the prognostic nomogram using population-based data to estimate the survival outcomes of patients with NECC. The developed nomogram maintained high predictive accuracy, which could facilitate clinicians to make appropriate assessments. Future studies should aim to further validated this nomogram by a larger population.

## Supplementary Information

**Additional file 1:** **Figure S1.** The graph shows the optimal cut-off points of age via the X-tile program. The black dot demonstrates the best cut-off of age (A); the histogram and survival curves were represented based on cut-off points (B, C). The best cut-off points of age were 44 and 67 years

**Additional file 2:** **Table S1.** Demographics and Clinicopathologic Characterisitcs of patients with NECC form the Wenling Maternal and Child Health Care Hospital dataset

**Additional file 3:** **Figure S2.** The calibration curves of external validation cohort show the nomograms-predicted rates (X-axis) are correspondent with the actual survival rates (Y-axis), including the 3-year CSS (A) and OS (B), and the 5-year CSS (C) and OS (D)

## Data Availability

The datasets used and/or analyzed during the current study are available from the corresponding author on reasonable request.

## Abbreviations

NECC: Neuroendocrine cervical carcinoma
OS: Overall survival
CSS: Cancer-specific survival
ROC: Receiver operating characteristic
FIGO: The International Federation of Gynecology and Obstetrics
CIF: The cumulative incidence function

## References

[1] Cohen JG, Kapp DS, Shin JY, Urban R, Sherman AE, Chen LM, Osann K, Chan JK (2010). Small cell carcinoma of the cervix: treatment and survival outcomes of 188 patients. Am J Obstet Gynecol.

[2] Margolis B, Tergas AI, Chen L, Hou JY, Burke WM, Hu JC, Ananth CV, Neugut AI, Hershman DL, Wright JD (2016). Natural history and outcome of neuroendocrine carcinoma of the cervix. Gynecol Oncol.

[3] Gadducci A, Carinelli S, Aletti G (2017). Neuroendrocrine tumors of the uterine cervix: A therapeutic challenge for gynecologic oncologists. Gynecol Oncol.

[4] Burzawa J, Gonzales N, Frumovitz M (2015). Challenges in the diagnosis and management of cervical neuroendocrine carcinoma. Expert Rev Anticancer Ther.

[5] Castle PE, Pierz A, Stoler MH (2018). A systematic review and meta-analysis on the attribution of human papillomavirus (HPV) in neuroendocrine cancers of the cervix. Gynecol Oncol.

[6] Zatelli MC, Guadagno E, Messina E, Lo CF, Faggiano A, Colao A (2018). NIKE Group: **Open issues on G3 neuroendocrine neoplasms: back to the future**. Endocr Relat Cancer.

[7] Tempfer CB, Tischoff I, Dogan A, Hilal Z, Schultheis B, Kern P, Rezniczek GA (2018). Neuroendocrine carcinoma of the cervix: a systematic review of the literature. BMC Cancer.

[8] Lee DY, Chong C, Lee M, Kim JW, Park NH, Song YS, Park SY (2016). Prognostic factors in neuroendocrine cervical carcinoma. Obstet Gynecol Sci.

[9] Salvo G, Gonzalez MA, Gonzales NR, Frumovitz M (2019). Updates and management algorithm for neuroendocrine tumors of the uterine cervix. Int J Gynecol Cancer.

[10] Elsherif S, Odisio E, Faria S, Javadi S, Yedururi S, Frumovitz M, Ramalingam P, Bhosale P (2018). Imaging and staging of neuroendocrine cervical cancer. Abdom Radiol (NY).

[11] Nagao S, Miwa M, Maeda N, Kogiku A, Yamamoto K, Morimoto A, Wakahashi S, Ichida K, Sudo T, Yamaguchi S (2015). Clinical Features of Neuroendocrine Carcinoma of the Uterine Cervix: A Single-Institution Retrospective Review. Int J Gynecol Cancer.

[12] Ning L, Zhang W, Yang J, Cao D, Lang J, Yu M (2018). Prognostic factors of FIGO stage I-IIA small-cell neuroendocrine carcinoma of the uterine cervix. Int J Gynaecol Obstet.

[13] Iasonos A, Schrag D, Raj GV, Panageas KS (2008). How to build and interpret a nomogram for cancer prognosis. J Clin Oncol.

[14] Fang C, Wang W, Feng X, Sun J, Zhang Y, Zeng Y, Wang J, Chen H, Cai M, Lin J (2017). Nomogram individually predicts the overall survival of patients with gastroenteropancreatic neuroendocrine neoplasms. Br J Cancer.

[15] Lin Z, Wang H, Zhang Y, Li G, Pi G, Yu X, Chen Y, Jin K, Chen L, Yang S (2019). Development and Validation of a Prognostic Nomogram to Guide Decision-Making for High-Grade Digestive Neuroendocrine Neoplasms. Oncologist.

[16] Latouche A, Allignol A, Beyersmann J, Labopin M, Fine JP (2013). A competing risks analysis should report results on all cause-specific hazards and cumulative incidence functions. J Clin Epidemiol.

[17] Noordzij M, Leffondre K, van Stralen KJ, Zoccali C, Dekker FW, Jager KJ (2013). When do we need competing risks methods for survival analysis in nephrology?. Nephrol Dial Transplant.

[18] Gooley TA, Leisenring W, Crowley J, Storer BE (1999). Estimation of failure probabilities in the presence of competing risks: new representations of old estimators. Stat Med.

[19] van Walraven C, McAlister FA (2016). Competing risk bias was common in Kaplan-Meier risk estimates published in prominent medical journals. J Clin Epidemiol.

[20] Zaid T, Burzawa J, Basen-Engquist K, Bodurka DC, Ramondetta LM, Brown J, Frumovitz M (2014). Use of social media to conduct a cross-sectional epidemiologic and quality of life survey of patients with neuroendocrine carcinoma of the cervix: a feasibility study. Gynecol Oncol.

[21] Yang J, Li Y, Liu Q, Li L, Feng A, Wang T, Zheng S, Xu A, Lyu J (2020). Brief introduction of medical database and data mining technology in big data era. J Evid Based Med.

[22] Abdallah R, Bush SH, Chon HS, Apte SM, Wenham RM, Shahzad MM (2016). Therapeutic Dilemma: Prognostic Factors and Outcome for Neuroendocrine Tumors of the Cervix. Int J Gynecol Cancer.

[23] Chen J, Macdonald OK, Gaffney DK (2008). Incidence, mortality, and prognostic factors of small cell carcinoma of the cervix. Obstet Gynecol.

[24] Ishikawa M, Kasamatsu T, Tsuda H, Fukunaga M, Sakamoto A, Kaku T, Kato T, Takahashi K, Ariyoshi K, Suzuki K (2019). A multi-center retrospective study of neuroendocrine tumors of the uterine cervix: Prognosis according to the new 2018 staging system, comparing outcomes for different chemotherapeutic regimens and histopathological subtypes. Gynecol Oncol.

[25] Stecklein SR, Jhingran A, Burzawa J, Ramalingam P, Klopp AH, Eifel PJ, Frumovitz M (2016). Patterns of recurrence and survival in neuroendocrine cervical cancer. Gynecol Oncol.

[26] Xu F, Ma J, Yi H, Hu H, Fan L, Wu P, Chen X, Wu X, Yu L, Xing H (2018). Clinicopathological Aspects of Small Cell Neuroendocrine Carcinoma of the Uterine Cervix: a Multicenter Retrospective Study and Meta-Analysis. Cell Physiol Biochem.

[27] Robin TP, Amini A, Schefter TE, Behbakht K, Fisher CM (2016). Brachytherapy should not be omitted when treating locally advanced neuroendocrine cervical cancer with definitive chemoradiation therapy. Brachytherapy.

[28] Hou WH, Schultheiss TE, Wong JY, Wakabayashi MT, Chen YJ (2018). Surgery Versus Radiation Treatment for High-Grade Neuroendocrine Cancer of Uterine Cervix: A Surveillance Epidemiology and End Results Database Analysis. Int J Gynecol Cancer.

[29] Yuan L, Jiang H, Lu Y, Guo SW, Liu X (2015). Prognostic Factors of Surgically Treated Early-Stage Small Cell Neuroendocrine Carcinoma of the Cervix. Int J Gynecol Cancer.

[30] Chen TC, Huang HJ, Wang TY, Yang LY, Chen CH, Cheng YM, Liou WH, Hsu ST, Wen KC, Ou YC (2015). Primary surgery versus primary radiation therapy for FIGO stages I-II small cell carcinoma of the uterine cervix: A retrospective Taiwanese Gynecologic Oncology Group study. Gynecol Oncol.

[31] Castelnau-Marchand P, Pautier P, Genestie C, Leary A, Bentivegna E, Gouy S, Scoazec JY, Morice P, Haie-Meder C, Chargari C (2018). Multimodal Management of Locally Advanced Neuroendocrine Cervical Carcinoma: A Single Institution Experience. Int J Gynecol Cancer.

[32] McCann GA, Boutsicaris CE, Preston MM, Backes FJ, Eisenhauer EL, Fowler JM, Cohn DE, Copeland LJ, Salani R, O'Malley DM (2013). Neuroendocrine carcinoma of the uterine cervix: the role of multimodality therapy in early-stage disease. Gynecol Oncol.

[33] Frumovitz M, Munsell MF, Burzawa JK, Byers LA, Ramalingam P, Brown J, Coleman RL (2017). Combination therapy with topotecan, paclitaxel, and bevacizumab improves progression-free survival in recurrent small cell neuroendocrine carcinoma of the cervix. Gynecol Oncol.

[34] Sharabi A, Kim SS, Kato S, Sanders PD, Patel SP, Sanghvi P, Weihe E, Kurzrock R (2017). Exceptional Response to Nivolumab and Stereotactic Body Radiation Therapy (SBRT) in Neuroendocrine Cervical Carcinoma with High Tumor Mutational Burden: Management Considerations from the Center For Personalized Cancer Therapy at UC San Diego Moores Cancer Center. Oncologist.

[35] Spigel DR, Waterhouse DM, Lane S, Legenne P, Bhatt K (2013). Efficacy and safety of oral topotecan and bevacizumab combination as second-line treatment for relapsed small-cell lung cancer: an open-label multicenter single-arm phase II study. Clin Lung Cancer.

[36] Lyons YA, Frumovitz M, Soliman PT (2014). Response to MEK inhibitor in small cell neuroendocrine carcinoma of the cervix with a KRAS mutation. Gynecol Oncol Rep.

