# Supplementary figures and images for: Construction and validation of the prognostic model for patients with neuroendocrine cervical carcinoma: a competing risk nomogram analysis
Source: BMC Cancer. 2022 Jan 3;22:4. doi: 10.1186/s12885-021-09104-9 (PMC8722105; doi:10.1186/s12885-021-09104-9)

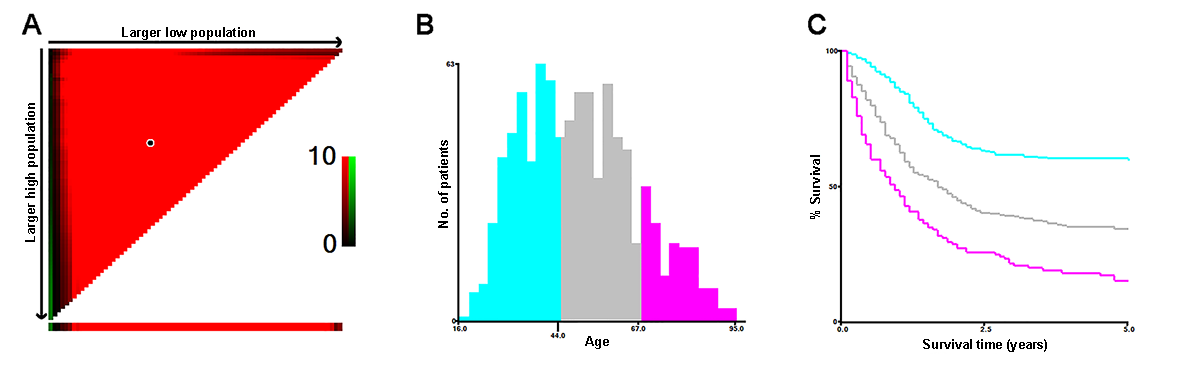

Supplement: Supplementary file 1 — Additional file 1: Figure S1. The graph shows the optimal cut-off points of age via the X-tile program. The black dot demonstrates the best cut-off of age (A); the histogram and survival curves were represented based on cut-off points (B, C). The best cut-off points of age were 44 and 67 years [file 12885_2021_9104_MOESM1_ESM.tif]

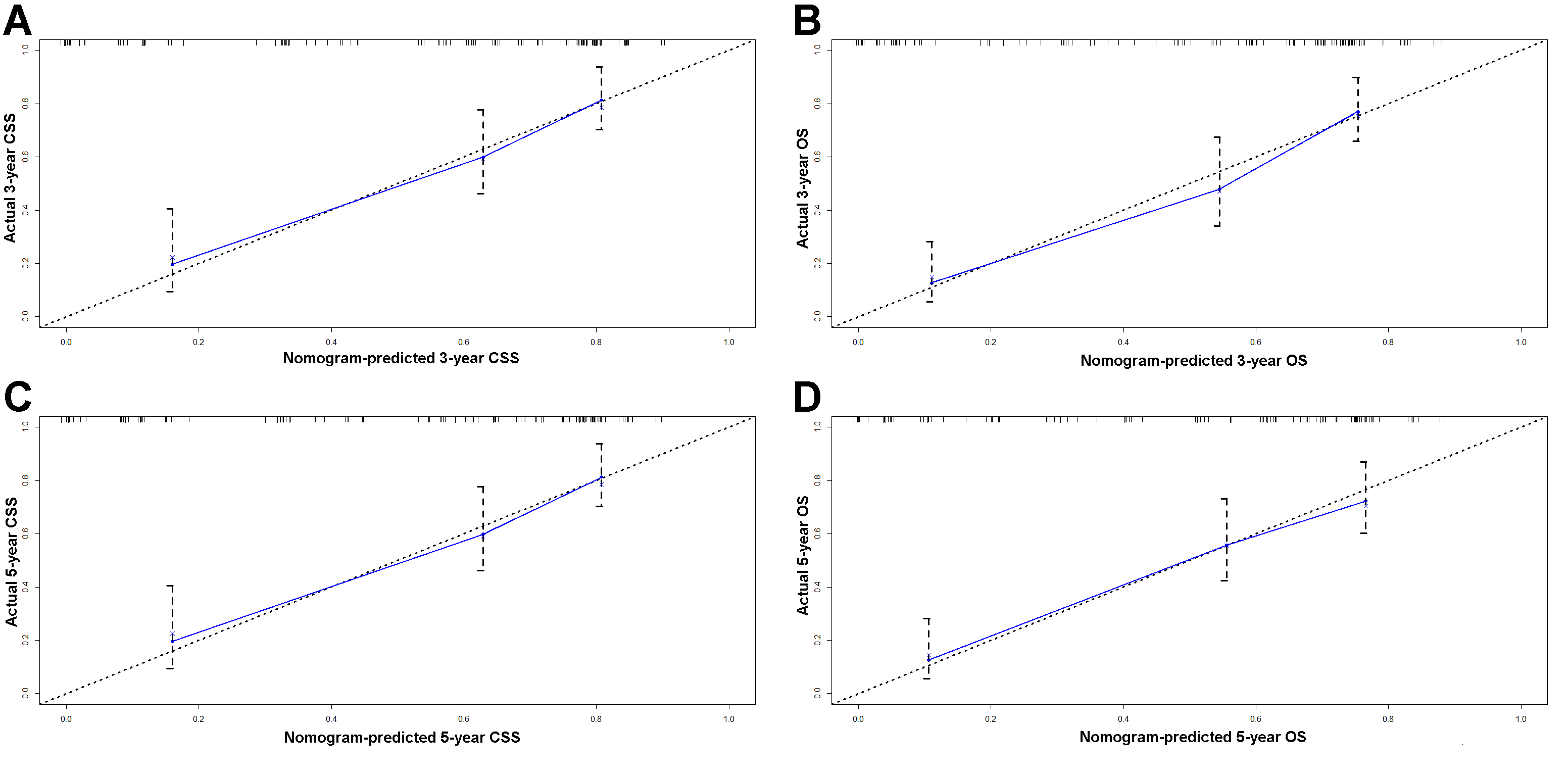

Supplement: Supplementary file 3 — Additional file 3: Figure S2. The calibration curves of external validation cohort show the nomograms-predicted rates (X-axis) are correspondent with the actual survival rates (Y-axis), including the 3-year CSS (A) and OS (B), and the 5-year CSS (C) and OS (D) [file 12885_2021_9104_MOESM3_ESM.tif]
